# The Medical Practitioner’s and Student’s Library

**Published:** 1848-04

**Authors:** 


*The Medical Prctitioner's and Student's Library*.—Under this title Messrs. Lindsay and Blakiston, of Philadelphia, propose to issue a series of original works on the various departments of Medical Science. Two volumes of the series have already appeared. One entitled "*Elements of the Principles and Practice of Midwifery*," by Daniel H. Tucker, M. D.; the other "*Elements of General Pathology*," by Alfred Stille, M. D. These volumes are to be followed speedily by a work on the diseases of children, from the pen of John F. Meigs, M. D.; a treatise on clinical medicine, by

Meredith Clymer; M. D., and a work on general or microscopical anatomy, by Francis G. Smith, M. D.

The following works are in preparation, to be comprised in the series:—On Minor Surgery, by Edward Hartshorne, M. D.; Special Anatomy, by John Neil, M. D.; Medical Chemistry, by Robert Bridges, M. D.; Materia Medica and Therapeutics, by Francis West, M. D.; Clinical Surgery, by Edward Hartshone, M. D.; and the American Dissector, by J. M. Allen, M. D. The price of each volume will be $1,25, or five volumes for $5. The books can be forwarded by mail; orders to be addressed to the Publishers.

We regard this enterprise as constituting an era in the history of the medical literature of this country. It is an important step toward the institution of a national medical literature. As such, it should meet with encouragement from the medical profession of the country. As Americans we should feel interested in it, although we do not doubt that its intrinsic claims should warrant success. The publishers deserve great credit, and we sincerely hope they will receive something more than empty praises. We shall notice in our next number, the volumes which have already appeared, and those which are to follow, as they are received.

